# Impact of diagnosis to treatment interval on outcomes in patients with newly diagnosed marginal zone lymphoma - a US multisite study

**DOI:** 10.1186/s40164-025-00666-z

**Published:** 2025-05-14

**Authors:** Narendranath Epperla, Geoffrey Shouse, Natalie S. Grover, Pallawi Torka, Kaitlin Annunzio, Marcus Watkins, Andrea Anampa-Guzmán, Beth Christian, Colin Thomas, Stefan K. Barta, Praveen Ramakrishnan Geethakumari, Reem Karmali, Nancy L. Bartlett, Adam J. Olszewski

**Affiliations:** 1 Division of Hematology, Ohio State University Comprehensive Cancer Center, Columbus, OH USA; 2 Division of Hematology and Hematologic Malignancies, Huntsman Cancer Institute, University of Utah, Salt Lake City, UT 84103 USA; 3 Department of Medicine, City of Hope National Medical Center, Duarte, CA USA; 4 Department of Medicine, Lineberger Comprehensive Cancer Center, University of North Carolina, Chapel Hill, NC USA; 5 Department of Medicine, Roswell Park Comprehensive Cancer Center, Buffalo, NY USA; 6 Department of Medicine, Memorial Sloan Kettering Cancer Center, New York, NY USA; 7 Department of Medicine, Siteman Cancer Center, Washington University School of Medicine, St. Louis, MO USA; 8 Department of Medicine, Thomas Jefferson University, Philadelphia, PA USA; 9 Department of Medicine, University of Pennsylvania, Philadelphia, PA USA; 10 Department of Medicine, Harold C. Simmons Comprehensive Cancer Center, UT Southwestern Medical Center, Dallas, TX USA; 11 Department of Medicine, Northwestern University, Chicago, IL USA; 12 Department of Medicine, Brown University Providence, Providence, RI USA

**Keywords:** DTI, MZL, Progression-free survival, Overall survival

## Abstract

Diagnosis-to-treatment interval (DTI) is an important prognostic factor in patients with newly diagnosed aggressive lymphomas, however the impact of DTI on outcomes in marginal zone lymphoma (MZL) is unknown. In this multicenter retrospective cohort study, we included adult patients with MZL who received first-line immunochemotherapy within 120 days of diagnosis at 10 US medical centers. Patients who received treatment within 60 days from their diagnosis were classified into the short DTI group and those who received treatment beyond 60 days into long DTI group. The primary objective was progression-free survival (PFS), while secondary objectives included overall survival (OS) and cumulative incidence of histologic transformation (HT) between the two groups. Of the 870 patients with newly diagnosed MZL, 177 patients met the inclusion criteria and were included in this analysis. Among these 144 (81%) were in the short DTI group and 33 (19%) in the long DTI group. In the univariable analysis, presence of B symptoms was associated with short DTI and remained significantly associated with short DTI in the multivariable analysis (OR = 11.91, p = 0.017). Short DTI was not associated with a statistically different PFS or OS compared to long DTI in the univariable or in multivariable analysis. The cumulative incidence of HT was not significantly different between the two groups. This is the first study to-date to report on the association of DTI on outcomes in MZL patients. This lack of prognostic utility of DTI in newly diagnosed MZL, in contrast to aggressive B-cell lymphomas, may be intrinsically linked to the underlying disease biology.

**Supplementary Information:**

The online version contains supplementary material available at 10.1186/s40164-025-00666-z.

## To the editor

Diagnosis to treatment interval (DTI) is an important prognostic factor in patients with newly diagnosed diffuse large B-cell lymphoma (DLBCL) and mantle cell lymphoma (MCL) [1, 2] where patients with short DTI (< 14 days) consistently show inferior survival. However, the impact of DTI in marginal zone lymphoma (MZL) is unknown.

In this multicenter retrospective cohort study, we included adult MZL patients who received first-line immunochemotherapy within 120 days of diagnosis at 10 US medical centers. In a sensitivity analysis, we observed no difference in the main results when varying this cutoff (Figure S1a, b). We collected variables known to be significantly associated with survival outcomes in all subtypes of MZL [3–6].

DTI was defined as the time in days from the date of diagnosis (biopsy confirmed) to the initiation of immunochemotherapy. Patients who received treatment within 60 days from their diagnosis were classified into short DTI group and those who received treatment > 60 days into long DTI group. Primary objective was evaluation of progression-free survival (PFS) while secondary objectives included overall survival (OS) and cumulative incidence of transformation between the two groups. For details on methods including statistical analysis, see Table S1.

Of the 870 patients with newly diagnosed MZL, 177 patients met the inclusion criteria (CONSORT diagram, Figure S2). Among these, 144 (81%) were in the short DTI group. Median age was 61 years with 43% EMZL (n = 77), 38% NMZL (n = 67), and 19% SMZL (n = 33) patients. 75% received bendamustine and rituximab (BR) as first-line treatment followed by rituximab, cyclophosphamide, doxorubicin, vincristine, prednisone (R-CHOP, 15%), and rituximab, cyclophosphamide, vincristine, prednisone (R-CVP, 10%). Table 1 shows the baseline characteristics of the patient population according to DTI group.

**Table 1:** Baseline characteristics

	All patients N = 177 (%)	DTI ≤ 60 days n = 144 (%)	DTI > 60 days n = 33 (%)
Median age in years (range)	61 (21–91)	61 (21–91)	61 (42–82)
Gender			
Male	84 (48)	70 (49)	14 (42)
Female	93 (52)	74 (51)	19 (58)
Race			
White	129 (75)	105 (76)	24 (73)
African American	30 (18)	25 (18)	5 (15)
Others	12 (7)	8 (6)	4 (12)
MZL subtype			
NMZL	67 (38)	54 (38)	13 (39)
SMZL	33 (19)	29 (20)	4 (12)
EMZL	77 (43)	61 (42)	16 (49)
ECOG PS			
0–1	142 (92)	114 (92)	28 (93)
≥ 2	12 (8)	10 (8)	2 (7)
Stage			
1–2	18 (10)	12 (8)	6 (18)
3–4	159 (90)	132 (92)	27 (82)
BM involvement			
No	57 (40)	41 (36)	16 (57)
Yes	85 (60)	73 (64)	12 (43)
Not done	35	30	5
B symptoms			
No	132 (77)	101 (73)	31 (97)
Yes	39 (23)	38 (27)	1 (3)
LDH > ULN			
No	115 (74)	90 (73)	25 (78)
Yes	41 (26)	34 (27)	7 (22)
Ki67 > 20%			
No	73 (71)	58 (72)	15 (68)
Yes	30 (29)	23 (28)	7 (32)
Not tested	44	63	11
Monoclonal paraprotein			
No	55 (57)	38 (3)	17 (71)
Yes	42 (43)	35 (48)	7 (29)
Not tested	80	71	9
WBC (K/uL), median (range)	6.1 (0.7–151)	5.9 (0.7–151)	6.4 (2.5–90.6)
Hb (g/dL), median (range)	11.9 (3.7–16.1)	11.8 (3.7–16.1)	12.4 (7.7–15.8)
Serum albumin			
Normal	135 (85)	109 (86)	26 (81)
Low^	23 (15)	17 (14)	6 (19)
First-line therapy			
BR	132 (75)	106 (74)	26 (79)
RCHOP	27 (15)	23 (16)	4 (12)
RCVP	18 (10)	15 (10)	3 (9)

*NMZL* Nodal marginal zone lymphoma, *SMZL* Splenic marginal zone lymphoma, *EMZL* Extranodal marginal zone lymphoma, *ECOG PS* Eastern Cooperative Oncology Group Performance Status, *BM* bone marrow, *LDH* Lactate Dehydrogenase, *ULN* Upper limit of normal, *WBC* White blood cell, *BR* rituximab, bendamustine, *R-CHOP* rituximab, cyclophosphamide, doxorubicin, vincristine, prednisone, *RCVP* rituximab, cyclophosphamide, vincristine, prednisone, *DTI* Diagnosis to treatment interval

*Percentages based on total number of patients with available data

^based on institutional standard

In the univariable analysis, presence of B symptoms was associated with short DTI (Table S2) and remained significantly associated with short DTI in the multivariable analysis after controlling for other clinically relevant factors (OR = 11.91, p = 0.017, Table S2).

**Fig. 1:**
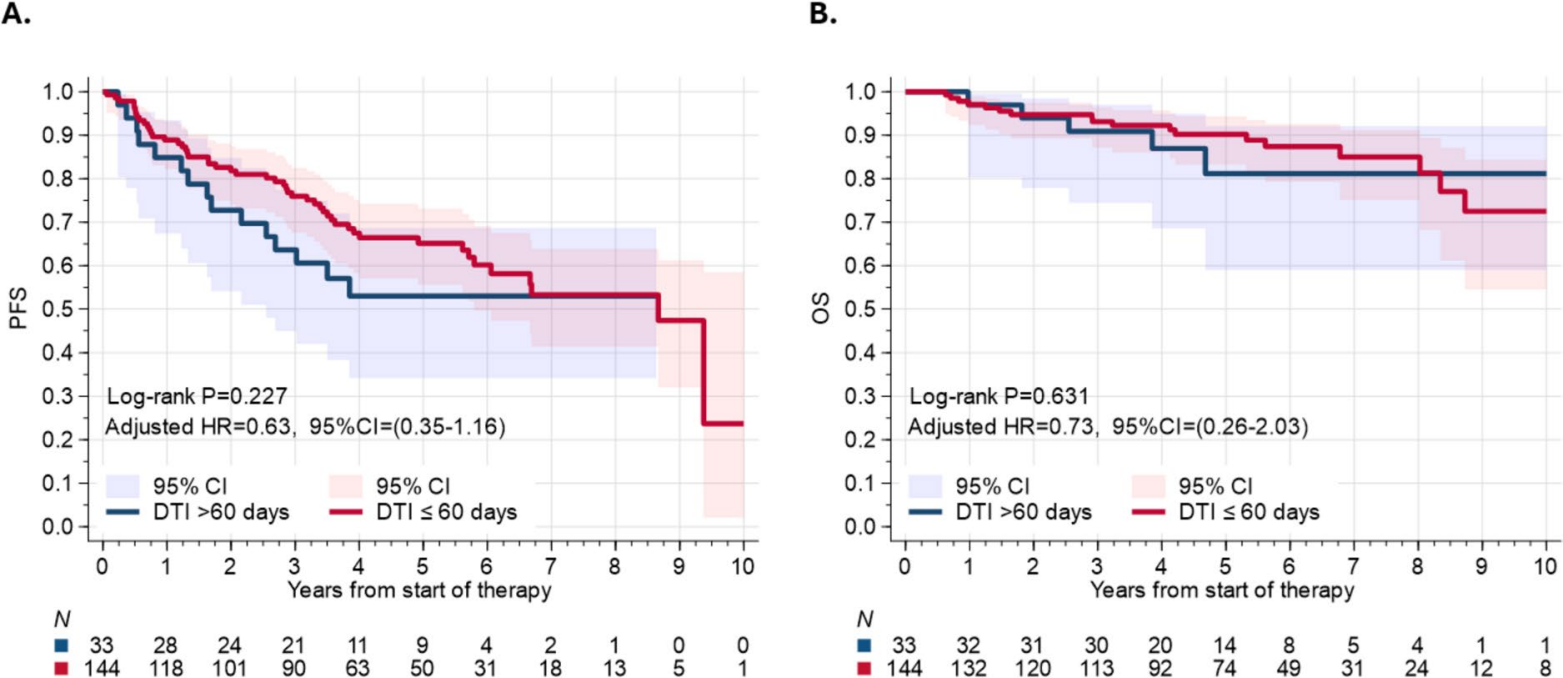
Association between the DTI and survival in newly diagnosed patients with marginal lymphoma treated with immunochemotherapy. **a** Progression-free survival **b** Overall survival

Short DTI was not associated with a statistically different PFS compared to long DTI in univariable assessment (HR = 0.70, 95% CI = 0.39–1.25, p = 0.22), or in the multivariable analysis, (aHR = 0.63, 95% CI = 0.35–1.16, p = 0.14, Fig. 1a). In the multivariable model (Table S3), factors independently associated with significantly inferior PFS included advancing age (aHR = 1.28, 95% CI = 1.02–1.61, p = 0.035) and low albumin at diagnosis (aHR = 2.40, 95% CI = 1.19–4.83, p = 0.01), while patients who received BR had significantly better PFS compared to those who received R-CHOP/RCVP (aHR = 0.29, 95% CI = 0.17–0.51, p < 0.001, Figure S3a).

Short DTI was not associated with inferior OS compared to long DTI in either univariable assessment (HR = 0.78, 95% CI = 0.29–2.12, p = 0.63, Fig. 1b) or multivariable analysis (aHR = 0.73, 95% CI = 0.26–2.03, p = 0.55). In the multivariable model (Table S4), we did not identify any factors associated with significantly inferior OS including receipt of BR (Figure S3b), although advancing age trended towards inferior OS (aHR = 1.53, 95% CI = 0.99–2.36, p = 0.05, Table S4).

There was no evidence of statistically significant interaction between DTI and histologic MZL subtype for PFS (interaction *P* = 0.88) or OS (*P* = 0.53). The main results were not sensitive to the choice of specific DTI cutoff (ranging from 20 to 100 days) for group discrimination (Figure S4).

There were 12 transformation events in the study, 8 in the short DTI group and 4 in the long DTI group. The cumulative incidence of transformation was not significantly different between the two groups (10-year CIF 13% in the short DTI group vs. 12% in the long DTI group, p = 0.22, Figure S5).

In this multicenter retrospective cohort study evaluating the impact of DTI on outcomes in newly diagnosed MZL treated with immunochemotherapy, we made several important observations. First, a short DTI does not correlate with an inferior PFS, despite being associated with certain unfavorable characteristics such as the presence of B symptoms. Second, short DTI does not portend inferior OS. Lastly, there was no difference in the cumulative incidence of transformation between short or longer DTI.

We included patients with MZL treated with immunochemotherapy to avoid heterogeneity related to first-line treatment (such as those treated with rituximab monotherapy) and avoid treatment bias. Despite being associated with high-risk factors, short DTI was not associated with inferior survival, which is in contrast to the literature in aggressive lymphomas (DLBCL and MCL) [1, 2]. This may be intrinsically linked to the differences in disease biology and future studies need to explore if there are any high-risk groups within MZL that may behave differently with regard to DTI. Furthermore, the observed improvement in PFS with BR compared to RCHOP/RCVP, as demonstrated in clinical trials like STiL [7] and BRIGHT [8], cannot be attributed to differences in DTI.

## Supplementary Information

Additional file 1.

## Data Availability

No datasets were generated or analysed during the current study.
