# Supplementary material for: Impact of diagnosis to treatment interval on outcomes in patients with newly diagnosed marginal zone lymphoma - a US multisite study
Source: Exp Hematol Oncol. 2025 May 14;14:73. doi: 10.1186/s40164-025-00666-z (PMC12079943; doi:10.1186/s40164-025-00666-z)

**SUPPLEMENTAL APPENDIX**

**Table of Contents** 1

**Methods:** Table S1 2

**Factors associated with short DTI:** Table S2 3

**Factors prognostic of PFS in multivariable analysis:** Table S3 4

**Factors prognostic of OS in multivariable analysis:** Table S4 5

**Cumulative distribution of DTI and sensitivity analysis:** Figure S1 6

**CONSORT Diagram:** Figure S2 7

**Outcomes of first-line therapy in newly diagnosed MZL pts:** Figure S3 8

**Sensitivity analyses to the DTI cutoff:** Figure S4 9

**Cumulative incidence of transformation between 2 groups:** Figure S5 10

**Table S1. Methods**

| **Eligibility** |
| --- |
| Patients who never received systemic therapy or those who received only rituximab, antibiotics, radiation therapy, or surgery were excluded, as were those whose lymphoma was managed initially with watchful waiting (as evidenced by medical record or a DTI >120 days). |
| **Variables of interest** |
| Values of laboratory tests (albumin, hemoglobin, and serum lactate dehydrogenase [LDH]) were harmonized according to the upper or lower limit of normal at each institution, and missing data were accounted for using multiple imputation by chained equations as previously described. All staging procedures (e.g., bone marrow evaluations) and treatment evaluations were conducted according to local practice. |
| **Definitions** |
| PFS was defined as the time from the start of first-line therapy to lymphoma relapse, progression, death from any cause, or censoring at the last clinical assessment. OS was defined as the time from the start of first-line systemic therapy until death or last follow‐up. |
| **Statistical analysis** |
| Demographic and disease characteristics were summarized using medians and ranges for continuous variables and frequencies and percentages for categorical variables. They were compared among study groups using the rank sum test for continuous variables and Fisher exact test for categorical variables. To compare clinical characteristics between the two groups, univariable and multivariable logistic regression analyses were performed, accounting for missing data through multiple imputations. Using concordance statistics for different DTI cutoffs, we determined 60 days as optimal for analysis. PFS was estimated using the Kaplan-Meier method and compared between groups using the log-rank test. The cumulative incidence of histologic transformation was calculated by treating death as a competing risk and compared between groups using competing regression models. OS was calculated from the start of first-line treatment and compared using the log-rank test. Cox proportional hazard regression models were used to estimate the hazard ratios (HRs) for risk of progression or death. Analyses were performed using Stata version 18.0 (StataCorp, College Station, TX), and all statistical tests were 2-sided, with a type-1 error <0.05 indicating statistical significance. All estimates were reported with 95% confidence intervals (95% CIs). |

**Table S2: Factors associated with short DTI**

| **Variable** | **Univariable** | | **Multivariable** | |
| --- | --- | --- | --- | --- |
|  | **OR (95% CI)** | **p-value** | **OR (95% CI)** | **p-value** |
| Age | 0.96 (0.69-1.35) | 0.82 | 0.92 (0.62-1.36) | 0.68 |
| Gender |  |  |  |  |
| Male | Referent |  |  |  |
| Female | 0.78 (0.36-1.67) | 0.52 | 0.69 (0.29-1.64) | 0.41 |
| ECOG PS |  |  |  |  |
| 0-1 | Referent |  |  |  |
| ≥2 | 1.31 (0.27-6.32) | 0.74 | 0.79 (0.11-5.64) | 0.82 |
| MZL subtype |  |  |  |  |
| NMZL | Referent |  |  |  |
| SMZL | 1.83 (0.60-5.61) | 0.29 | 1.57 (0.40-6.10) | 0.52 |
| EMZL | 0.78 (0.37-1.67) | 0.52 | 1.11 (0.45-2.76) | 0.82 |
| Stage |  |  |  |  |
| 1-2 | Referent |  |  |  |
| 3-4 | 1.62 (0.58-4.58) | 0.36 | 1.06 (0.33-3.39) | 0.93 |
| B-symptoms |  |  |  |  |
| No | Referent |  |  |  |
| Yes | 8.65 (1.24-60.08) | **0.029** | 11.91 (1.55-91.33) | **0.017** |
| LDH > ULN |  |  |  |  |
| No | Referent |  |  |  |
| Yes | 1.34 (0.53-3.40) | 0.53 | 0.90 (0.30-2.72) | 0.85 |
| Ki67>20% * |  |  |  |  |
| No | Referent |  |  |  |
| Yes | 0.85 (0.31-2.35) | 0.75 |  |  |
| Monoclonal paraprotein * |  |  |  |  |
| No | Referent |  |  |  |
| Yes | 2.24 (0.83-6.04) | 0.11 |  |  |
| WBC | 1.00 (0.97-1.02) | 0.80 | 1.00 (0.97-1.03) | 0.96 |
| Hgb | 0.84 (0.70-1.01) | 0.06 | 0.81 (0.63-1.04) | 0.09 |
| Albumin | 0.84 (0.31-2.30) | 0.74 | 0.27 (0.07-1.09) | 0.07 |
| Frontline Therapy |  |  |  |  |
| R-CHOP/R-CVP | Referent |  |  |  |
| BR | 0.75 (0.30-1.87) | 0.53 | 0.77 (0.28-2.15) | 0.62 |

Abbreviations: NMZL- Nodal marginal zone lymphoma, SMZL- Splenic marginal zone lymphoma, EMZL- Extranodal marginal zone lymphoma, ECOG PS- Eastern Cooperative Oncology Group Performance Status, LDH- Lactate Dehydrogenase, ULN- Upper limit of normal, WBC- White blood cell, BR- rituximab, bendamustine, R-CHOP- rituximab, cyclophosphamide, doxorubicin, vincristine, prednisone, RCVP- rituximab, cyclophosphamide, vincristine, prednisone

* Only univariate analysis using complete cases conducted—the multiple imputation model could not converge due to high proportion of missing data.

**Table S3: Factors prognostic of PFS in multivariable analysis**

| **Variable** | **HR (95% CI)** | **p-value** |
| --- | --- | --- |
| DTI |  |  |
| > 60 days | Referent |  |
| ≤ 60 days | 0.63 (0.35, 1.16) | 0.14 |
| Age | 1.28 (1.02, 1.61) | **0.035** |
| Gender |  |  |
| Male | Referent |  |
| Female | 0.95 (0.56, 1.87) | 0.95 |
| ECOG PS |  |  |
| 0-1 | Referent |  |
| ≥2 | 0.70 (0.19-2.59) | 0.59 |
| MZL subtype |  |  |
| NMZL | Referent |  |
| SMZL | 1.61 (0.82, 3.14) | 0.16 |
| EMZL | 1.02 (0.56, 1.87) | 0.95 |
| Stage |  |  |
| 1-2 | Referent |  |
| 3-4 | 1.69 (0.64, 4.49) | 0.29 |
| Serum albumin |  |  |
| Normal | Referent |  |
| Low | 2.40 (1.19, 4.83) | **0.01** |
| LDH > ULN |  |  |
| No | Referent |  |
| Yes | 1.02 (0.52, 1.98) | 0.96 |
| Frontline Therapy |  |  |
| R-CHOP/R-CVP | Referent |  |
| BR | 0.29 (0.17, 0.51) | **<0.001** |

Abbreviations: NMZL- Nodal marginal zone lymphoma, SMZL- Splenic marginal zone lymphoma, EMZL- Extranodal marginal zone lymphoma, ECOG PS- Eastern Cooperative Oncology Group Performance Status, LDH- Lactate Dehydrogenase, ULN- Upper limit of normal, BR- rituximab, bendamustine, R-CHOP- rituximab, cyclophosphamide, doxorubicin, vincristine, prednisone, RCVP- rituximab, cyclophosphamide, vincristine, prednisone, DTI- Diagnosis to treatment interval

**Table S4: Factors prognostic of OS in multivariable analysis**

| **Variable** | **HR (95% CI)** | **p-value** |
| --- | --- | --- |
| DTI |  |  |
| > 60 days | Referent |  |
| ≤ 60 days | 0.73 (0.26, 2.03) | 0.54 |
| Age | 1.53 (0.99, 2.36) | 0.05 |
| Gender |  |  |
| Male | Referent |  |
| Female | 1.47 (0.60, 3.63) | 0.40 |
| ECOG PS |  |  |
| 0-1 | Referent |  |
| ≥2 | 0.27 (0.03-2.28) | 0.23 |
| MZL subtype |  |  |
| NMZL | Referent |  |
| SMZL | 1.65 (0.56, 4.88) | 0.36 |
| EMZL | 0.93 (0.33, 2.63) | 0.89 |
| Stage |  |  |
| 1-2 | Referent |  |
| 3-4 | 0.95 (0.24, 3.82) | 0.94 |
| Serum albumin |  |  |
| Normal | Referent |  |
| Low | 2.80 (0.94, 8.38) | 0.06 |
| LDH > ULN |  |  |
| No | Referent |  |
| Yes | 1.04 (0.34, 3.23) | 0.94 |
| Frontline Therapy |  |  |
| R-CHOP/R-CVP | Referent |  |
| BR | 0.45 (0.18, 1.17) | 0.10 |

Abbreviations: NMZL- Nodal marginal zone lymphoma, SMZL- Splenic marginal zone lymphoma, EMZL- Extranodal marginal zone lymphoma, ECOG PS- Eastern Cooperative Oncology Group Performance Status, LDH- Lactate Dehydrogenase, ULN- Upper limit of normal, BR- rituximab, bendamustine, R-CHOP- rituximab, cyclophosphamide, doxorubicin, vincristine, prednisone, RCVP- rituximab, cyclophosphamide, vincristine, prednisone, DTI- Diagnosis to treatment interval

**Figure S1. (A) Cumulative distribution of DTI or all patients starting first-line immunochemotherapy within 1 year from diagnosis; (B) Sensitivity analysis: main HR results for PFS and OS while varying the maximum acceptable cutoff of DTI for inclusion in the analysis**

| **A** | **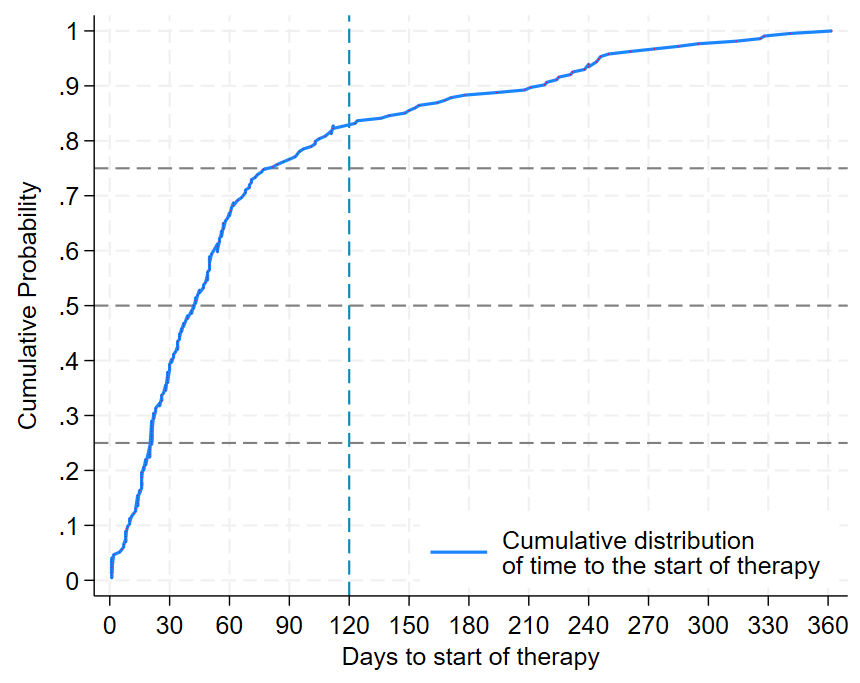** | **B** | 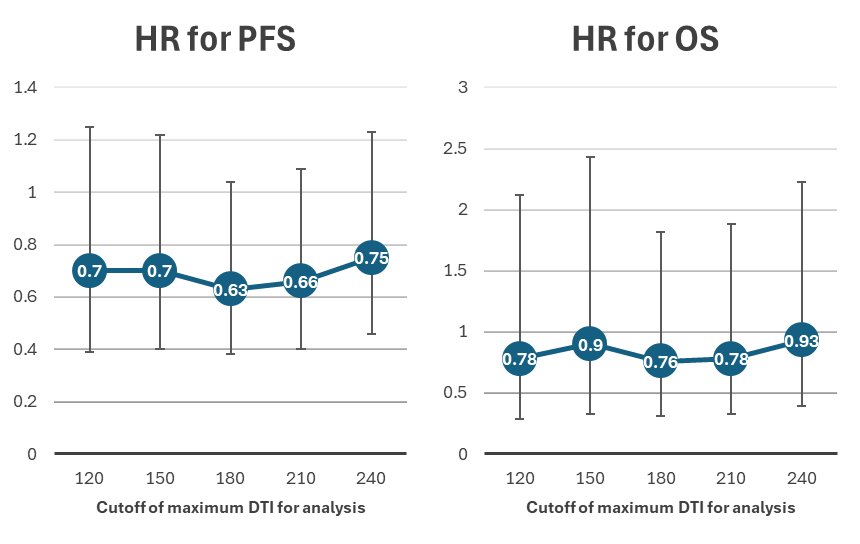 |
| --- | --- | --- | --- |

**Figure S2. CONSORT Diagram**

|  | **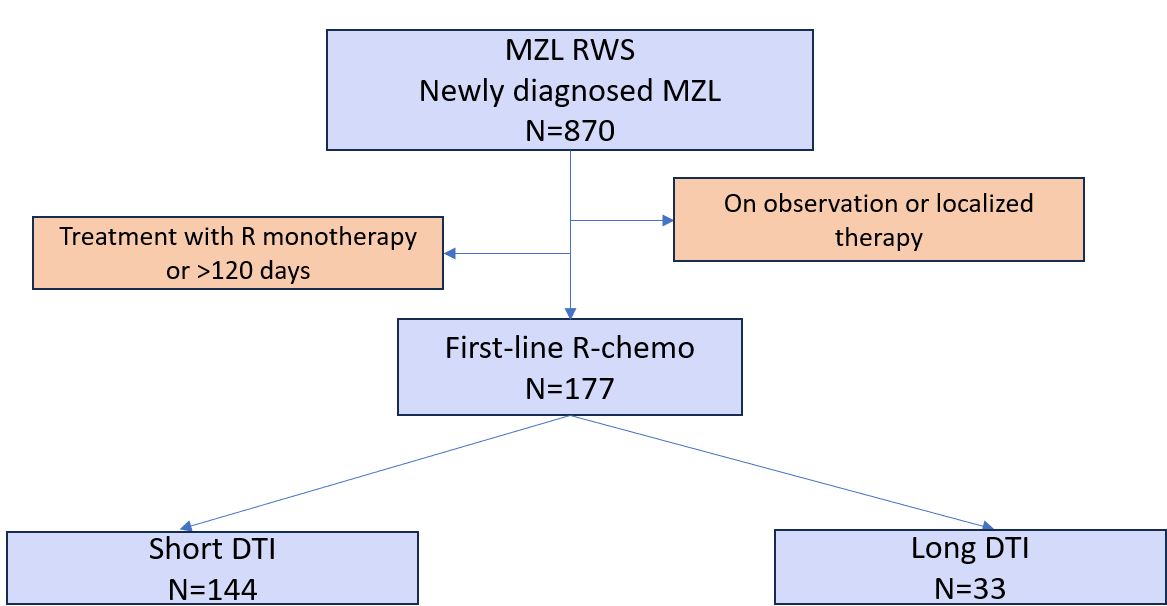** |
| --- | --- |

**Figure S3. Outcomes of first-line therapy in newly diagnosed patients with marginal zone lymphoma. A) Progression-free survival B) overall survival**

**Figure S4: Sensitivity analyses to the DTI cutoff.**

Plots show the observed (univariate) HR, as well as Harrell’s C statistics for the associated Cox model and the cumulative distribution of receipt of first-line immunochemotherapy for each DTI cutoff, which varied from 20 to 100 days. None of the models had shown a statistically significant result.

**Figure S5: Cumulative incidence of transformation between short DTI and long DTI in patients with newly diagnosed MZL treated with immunochemotherapy**


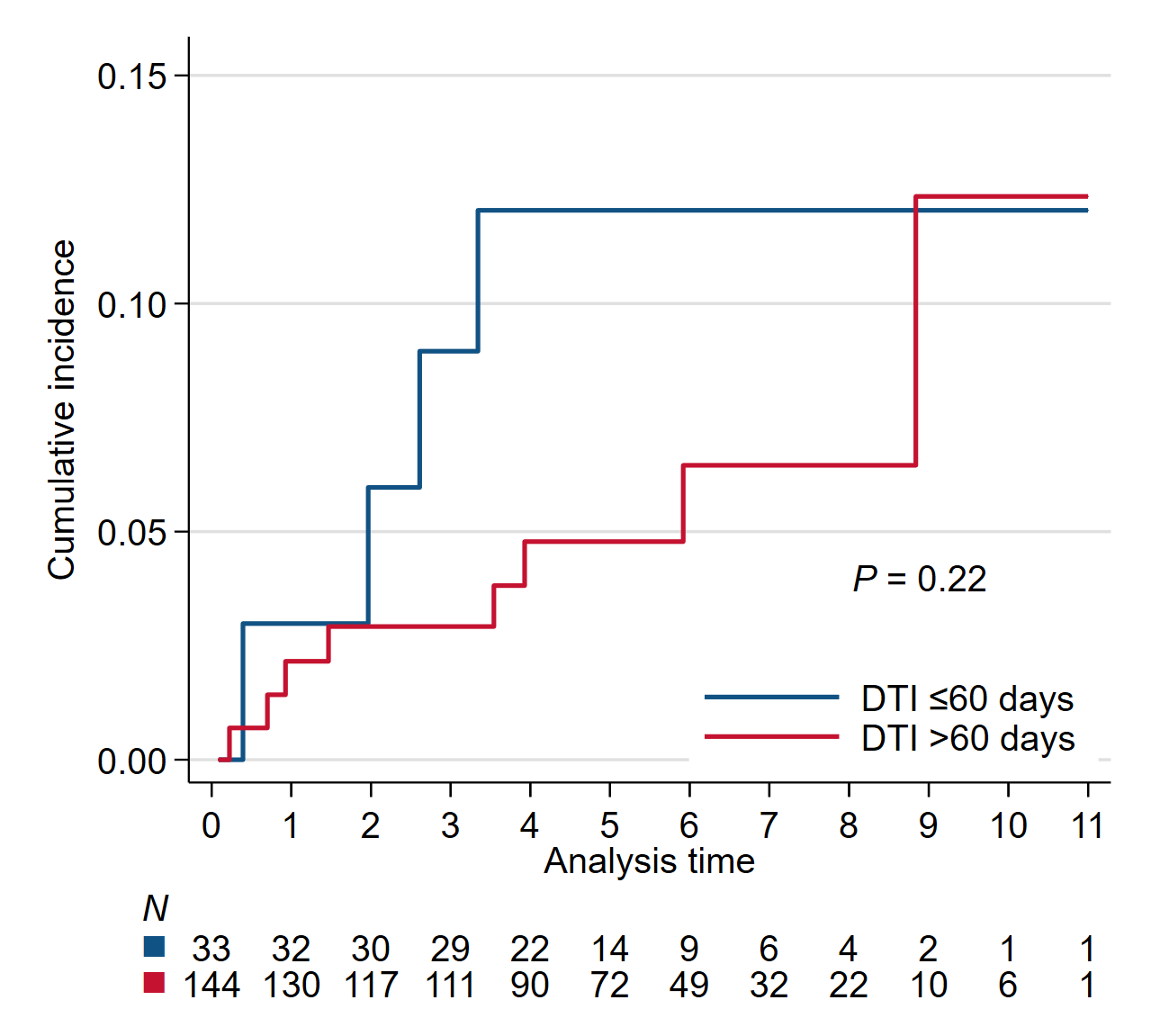

Supplement: Supplementary file 1 — Additional file 1. [file 40164_2025_666_MOESM1_ESM.docx]
